# Editorial: Long COVID in the biomedical sciences

**DOI:** 10.3389/bjbs.2026.17822

**Published:** 2026-10-09

**Authors:** Anil Philip Kunnath, Owen Kavanagh, Shing Cheng Tan

**Affiliations:** 1 Division of Applied Biomedical Science and Biotechnology, School of Health Sciences, IMU University, Bukit Jalil, Kuala Lumpur, Malaysia; 2 School of Health Sciences, York St John University, York, United Kingdom; 3 UKM Medical Molecular Biology Institute, Universiti Kebangsaan Malaysia, Cheras, Kuala Lumpur, Malaysia

**Keywords:** computed tomography (CT), long COVID symptoms, post-acute sequelae of SARS-CoV-2 infection (PASC), SARS-CoV 2, SARS-CoV-2 antibodies

With this special issue, the British Journal of Biomedical Science turns its attention to one of the most perplexing medical challenges of the post-pandemic era: Long COVID, or post-acute sequelae of SARS-CoV-2 infection (PASC). Millions worldwide continue to live with this condition, and health systems are only beginning to consider its long-term consequences. Manifestations of this condition are heterogeneous (e.g., debilitating fatigue, cognitive dysfunction, persistent respiratory compromise) affecting multiple organ systems. Despite intensive efforts, its underlying pathophysiological mechanisms remain elusive impeding the development of effective therapies. Against this background, this Special Issue brings together five original research articles addressing this problem from complementary perspectives, encompassing thoracic imaging, carotid body morphology, serological biomarkers, and microbiota-directed treatment. Read together, they advance a more coherent picture of its pathophysiology and highlight a shared premise: Long COVID cannot be understood through any single disciplinary lens.

That coherence begins with the assessment of persistent lung abnormalities. Garg et al. demonstrated that specific imaging patterns in the lungs of survivors of severe COVID-19 acute respiratory distress syndrome (ARDS) - such as reticulations/septal thickening–positively correlate with symptomatic dyspnoea. They also found that parenchymal/atelectatic bands were associated with reduced forced vital capacity. Taken together, these findings contribute to the current knowledge by demonstrating associations between individual CT imaging patterns and both symptomatic and functional measures, rather than merely documenting persistent structural abnormalities. A companion study in this issue by Devkota et al. reveals that ultra-low-dose CT (ULDCT) is comparable to standard-dose CT to accurately detect persistent lung abnormalities in patients who recovering from moderate to severe acute COVID-19 pneumonia. The authors reported a high ULDCT diagnostic accuracy of 95.0% (and 98% among non-obese subjects), making repeated surveillance imaging more feasible whilst reducing radiation exposure seen with standard methods.

Abnormalities associated with long COVID are not only confined to the lungs. Goksu et al. conducted a retrospective CT angiography study that identified significantly enlarged carotid bodies in patients previously hospitalized with COVID-19 pneumonia. As the carotid body plays a role in sensing blood hypoxemia and modulating sympathetic activity, this observation could reflect prolonged chemoreceptor stimulation resulting from pathophysiological changes in long COVID. Because the carotid body is rich in ACE2 receptors and central to chemoreception, this observation raises pointed questions about whether direct viral injury, chronic hypoxic stimulation, or both are at work, and it extends the syndrome’s reach well past the respiratory system.

Moving from structural abnormalities to immunological signals, Kawaguchi et al. investigated serological profiles to attempt to identify clinically useful biomarkers for Long COVID’s heterogeneous manifestations. In their study of 275 patients with Omicron-related Long COVID, nucleocapsid N-antibody titers were positively associated with acute disease severity and antiviral immune responses. Conversely, lower spike protein S-antibody titers correlated significantly with memory impairment (brain fog) and a reduced quality of life. These results expand our understanding of the immunological underpinnings of neurocognitive symptoms and may enhance the feasibility of a biomarker for this syndrome. The authors caution that factors such as vaccination status and time since infection must be carefully accounted for when interpreting serological profiles.

Finally, the issue turns from diagnosis to therapeutic intervention with a trial by Amoroso et al. In a randomized, double-blind, placebo-controlled study, the authors report that 4 weeks of a probiotic supplementation led to a significantly greater reduction in clinically relevant fatigue scores in patients with long COVID compared to placebo (24.24% vs. 6.06%). The concurrent changes in gut microbial taxa, microbiota-associated metabolites, and immune-related pathways offers a plausible mechanism for this clinical improvement. While the modest sample size warrants caution, this provides an exciting indication that gut-directed therapies deserve serious consideration in the Long COVID treatment landscape.

Taken as a whole, these five studies reach the same conclusion: Long COVID is a syndrome of genuine complexity. Continued research using diverse, integrated methodologies - including imaging, immunological, physiological, and microbiome-related measures - will be essential for improving the characterization, monitoring and treatment of long COVID.

We are grateful to the authors, reviewers, and editorial colleagues whose work has brought this issue together. Much remains unresolved in elucidating the underlying causes of Long COVID never mind alleviating the burden on patients and health systems. The British Journal of Biomedical Science remains committed to serving as a forum for the rigorous, multidisciplinary work this condition demands, and we look forward to continuing this vital quest with the research community.

